# Abrogation of KLF5 sensitizes *BRCA1*-proficient pancreatic cancer to PARP inhibition: KLF5 mediates sensitivity to PARP in *BRCA1*-proficient PDAC

**DOI:** 10.3724/abbs.2023288

**Published:** 2024-03-04

**Authors:** Zheng Zhang, Yuxin Liu, Yaolin Xu, Zijin Xu, Jinbin Jia, Yun Jin, Wenquan Wang, Liang Liu

**Affiliations:** 1 Department of Pancreatic Surgery Zhongshan Hospital Fudan University Shanghai 200032 China; 2 Cancer Center Zhongshan Hospital Fudan University Shanghai 200032 China; 3 Department of General Surgery Zhongshan Hospital Fudan University Shanghai 200032 China; 4Institute of Liver Diseases Shanxi Medical University Taiyuan 030001 China; 5 Department of General Surgery Qingpu Branch of Zhongshan Hospital Affiliated to Fudan University Shanghai 201700 China; 6 Department of Hepatobiliary and Pancreatic Surgery the First People’s Hospital of Yunnan Province the Affiliated Hospital of Kunming University of Science and Technology Kunming 650500 China

**Keywords:** pancreatic cancer, wild-type *BRCA*, BRCAness, KLF5, BRCA1

## Abstract

Poly ADP-ribose polymerase (PARP) inhibitor monotherapies are selectively effective in patients with pancreatic, breast, prostate, and ovarian cancers with *BRCA1* mutations. Cancer patients with more frequent wild-type *BRCA* show poor responses to PARP inhibitors. Moreover, patients who are initially sensitive to these inhibitors eventually respond poorly to drugs. In the present study, we discover that abrogation of Kruppel-like factor 5 (KLF5) significantly inhibits homologous recombination, which is the main mechanism for DNA double-stranded repair. Furthermore, the downregulation of KLF5 expression promotes the DNA damage induced by olaparib and significantly reduces the IC _50_ of the RARP inhibitor in pancreatic cancer cells. Overexpression of BRCA1 reverses the above effects caused by silencing of *KLF5*. Olaparib combined with a KLF5 inhibitor has an enhanced cytotoxic effect. Mechanistically, we identify *BRCA1* as a KLF5 target gene. BRCA1 is positively correlated with KLF5 in PDAC tissue. Our results indicate that inhibition of KLF5 may induce BRCAness in a larger pancreatic cancer subset with proficient BRCA. The combination of KLF5 inhibitors and PARP inhibitors provides a novel treatment strategy to enhance the sensitivity of BRCA1-proficient pancreatic cancer to PARP inhibitors.

## Introduction

There were 466,003 cancer-related deaths and 495,773 new cases of pancreatic cancer (PC) worldwide in 2020 [1]. It is urgent to develop novel treatment strategies beyond surgery and traditional chemotherapy. Strickler *et al*. [2] demonstrated that patients pretreated with KRAS p. G12C-mutated advanced PC responded robustly to sotorasib and showed good tolerance [2]. Pembrolizumab is recommended for unresectable or metastatic pancreatic ductal adenocarcinomas (PDAC) harboring defective DNA mismatch-repair-deficiency or microsatellite instability-high, which occur in 1%‒2% of PDAC cases. The PARP inhibitor olaparib has been approved for maintenance treatment of pretreated patients with metastatic PDAC with a germline BRCA mutation (4%‒7% of PDAC) [3]. However, PARP inhibitors confer little clinical benefit to most pancreatic cancer patients with wild-type BRCA1/2. Therefore, it is urgent to develop a novel strategy for expanding PARP inhibitors to BRCA-proficient PC.

Krüppel-factor 5 (KLF5) is a member of the KLF family and acts as a transcription factor that is frequently overexpressed in cancers [4]. KLF5 often functions as an oncogenic protein by regulating multiple target genes, such as *Slug* [5], *p27* [6], and *Cyclin D1* [7], thus regulating cell migration and proliferation. Hence, the upregulation of KLF5 has been demonstrated to promote the progression of various human malignancies, such as breast cancer [8], bladder cancer [9], colorectal cancer [10], gastric cancer [11], and hepatocellular carcinoma [12]. High expression of KLF5 is significantly correlated with a worse prognosis [12]. Emerging evidence has also revealed that KLF5 drives the progression of pancreatic cancer by inducing proliferation, glycolysis, and immunosuppression [ 13‒ 15]. We previously reported that FBW7 could regulate fibroblast growth factor (FGF)-binding protein-1 in a Myc-dependent way, which mediated the proliferation and migration of PDAC [16]. Emerging evidence has demonstrated that FBW7 acts as a vital tumor suppressor by targeting multiple oncogenic substrates in which the expression of KLF5, another key substrate, is also significantly regulated by FBW7 [17]. Li *et al*. [18] reported that KLF5 promoted the expression of Rad51, which could elicit DNA repair by homologous recombination. However, the role of KLF5 in PDAC DNA repair and sensitivity to PARP inhibitors remains unclear.

It has been demonstrated that homologous recombination repair (HRR) defects and PARP inhibition are synthetically lethal [19]. The tumor suppressor BRCA1/2 plays a key role in HRR [20]. Therefore, PARP inhibitors could significantly kill cancer cells with BRCA mutations. The inhibitor has been approved to treat patients with prostate, breast, and ovarian cancers with *BRCA* mutations based on several clinical trials [ 21‒ 23]. However, *BRCA* wild-type patients, accounting for the majority of all cancer patients, are not recommended for PARP inhibitors. Extensive subsequent studies have focused on expanding the use of these inhibitors to patients. A recent study showed that paclitaxel inhibits CDK1, resulting in weakened BRCA1 phosphorylation, which sensitizes ovarian cancer cells with proficient homologous recombination to PARP inhibitors [24]. Lu *et al*. [25] reported that inhibition of salt-inducible kinase 2 could produce synthetic lethality with several PARP inhibitors in homologous recombination DNA repair proficiency cancer cells by suppressing PARP enzyme activity and the DNA double-strand break repair pathway. Additionally, Ibrahim *et al*. [26] reported that suppression of PI3K inhibited BRCA expression and sensitive triple-negative breast cancer with proficient BRCA to PARP inhibition. In general, repression of BRCA expression or protein activity represents a promising strategy to broadly utilize PARP inhibitors in cancers.

In the present study, we explored the potential role of KLF5 in sensitivity to PARP inhibitors in pancreatic cancer cells. We discovered that repression of KLF5 elicited olaparib-induced DNA damage and significantly decreased the IC _50_ of olaparib in pancreatic cancer cells. Silencing of *KLF5* downregulated the expression of BRCA1 at the transcriptional level, which induced a “BRCAness”. Collectively, our results revealed a promising treatment target by which PARP inhibitors could be utilized to benefit a wider range of patients with pancreatic cancer.

## Materials and Methods

### Cell culture and small compounds

The human pancreatic duct epithelial cell line hTERT-HPNE and four PDAC cell lines PANC-1, CFPAC-1, BxPC3, and SW1990 were obtained from the American Type Culture Collection (ATCC; Manassas, USA) and cultured based on standard ATCC protocols. In short, hTERT-HPNE and PANC-1 cells were maintained in Dulbecco’s modified Eagle’s medium (DMEM; Gibco, Carlsbad, USA) with 10% fetal bovine serum (FBS; Gibco). BxPC3 cells were cultured in Roswell Park Memorial Institute (RPMI; Gibco) with 10% FBS. CFPAC-1 cells were cultured in Iscove’s modified Dulbecco’s medium containing 10% FBS (Gibco). SW1990 cells were maintained in Leibovitz (L-15; Gibco) with 10% FBS. In addition, 100 U/mL penicillin (Gibco) and 0.1 mg/mL streptomycin (Gibco) were added to all the culture media. SW1990 cells were cultured at 37°C in a humidified incubator without CO _2_. The remaining cells were maintained at 37°C in a humidified incubator with 5% CO _2_. Olaparib (HY-10162) was purchased from MedChemExpress (Monmouth Junction, USA).

### Quantitative real-time PCR (qRT-PCR)

Trizol reagent (Beyotime, Nantong, China) was utilized to isolate and purify total RNA. cDNAs were then obtained after reverse transcription using a PrimeScript RT reagent kit (TaKaRa, Dalian, China). Quantitative real-time PCR was performed as described previously [27]. All reactions were conducted in triplicate. The primers used in this study are presented in Supplementary Table S1.

### Western blot analysis

Standard western blot analysis was conducted. Total proteins from hTERT-HPNE and PDAC cells were extracted utilizing RIPA lysis buffer (Beyotime, Shanghai, China). The extracted proteins were electrophoresed in 10% SDS-PAGE gels and then transferred onto polyvinylidene difluoride membranes (Millipore, Billerica, USA). The membranes were incubated with primary antibodies overnight at 4°C and then incubated with the appropriate secondary antibody at room temperature for 1 h. An Enhanced chemiluminescence kit (Beyotime) was used to detect proteins. The antibodies used in this study were as follows: anti-β-actin (1:4000; Proteintech, Chicago, USA), anti-KLF5 (1:1000; Proteintech), anti-BRCA1 (1:1000; Proteintech), anti-Rad51 (1:1000; Proteintech), and HRP-conjugated secondary antibodies (1:3000; Proteintech).

### Plasmids

The TRC cloning vector (pLKO.1; Addgene, Cambridge, USA) was utilized to construct shRNA plasmids against KLF5 according to standard protocols [28]. Targets (21 bp) against *KLF5* were 5′-CCTATAATTCCAGAGCATAAA-3′ and 5′-GCTGTAATGTATATGGCTTTA-3′. pLKO.1-shKLF5, psPAX2, and pMD2.G were cotransfected into HEK-293T cells at a ratio of 4:3:1 to generate lentiviral particles. The coding sequences of human BRCA1 were cloned and inserted into the lentiviral vector pCDH-CMV-MCS-EF1-puro (SBI, San Francisco, USA) to generate BRCA1 expression plasmids.

### Cell viability assay

Cell Counting Kit-8 (CCK-8; Dojindo Laboratories, Tokyo, Japan) was used to investigate cell viability and cytotoxicity, as previously described [29].

### Immunohistochemical staining (IHC)

Clinical tissue samples were obtained from patients diagnosed with pancreatic cancer at Zhongshan Hospital, Fudan University. We obtained the patient’s consent and approval from the Institutional Research Ethics Committee of Zhongshan Hospital, Fudan University. Antibodies against KLF5 and BRCA1 were utilized to perform IHC in paraffin-embedded specimens of tissue according to standard IHC procedures. Anti-KLF5 antibody (Proteintech) and anti-BRCA1 antibody (Proteintech) were used at a dilution factor of 1:400. Positive intensity and proportion were scored as previously described [30].

### Colony formation assay

The cells were cultivated in 6-well plates at 500 cells per well for 10 days. The cells were then fixed utilizing 4% paraformaldehyde and stained with 0.1% crystal violet. Finally, the number of colonies was counted under a microscope (Olympus, Tokyo, Japan).

### DNA damage detection

To confirm whether DNA is damaged, a DNA Damage Assay kit (Beyotime) was used to detect the DNA damage markers γ-H2AX ( *i*. *e*., phosphorylated H2AX) according to the manufacturer’s instructions [31]. The images were taken by a confocal fluorescence microscope (Olympus).

### Dual-luciferase assay

The *BRCA1* promoter region from ‒2000 to +250 of the transcription start site or its related mutant sequence was cloned and inserted into the pGL3-Basic vector. The firefly and *Renilla* luciferase activities were measured using the Dual Luciferase Assay kit (Promega, Madison, USA), as previously described [32].

### Chromatin immunoprecipitation assay (ChIP)

The ChIP assays were conducted using the EZ-ChIP kit (Millipore), as previously described [33]. Primers to detect BRCA1 promoter occupancy are listed in Supplementary Table S1.

### Animal studies

Five-week-old male nude mice were purchased from the Shanghai SLAC Laboratory (Shanghai, China). About 6×10 ^6^ cells were subcutaneously inoculated into the left flank of the mice until the tumor volume reached ~100 mm ^3^. The mice were randomly divided into four subgroups (5 mice in each group): DMSO, olaparib, ML264, and a combination of olaparib and ML264. Intraperitoneal injections of olaparib (50 mg/kg) or ML264 (20 mg/kg) were administered daily. Next, tumor size was measured every 3 days and the tumor volume was calculated as length×width ^2^×0.5. At 5 weeks post-implantation, the tumor samples were surgically dissected. The protocol was approved by the Committee on the Ethics of Animal Experiments of Fudan University and conformed to the Guide for the Care and Use of Laboratory Animals published by the National Institutes of Health.

### Statistical analysis

Experiments were repeated at least three times. All statistical analyses were conducted using SPSS version 19.0 software (IBM) or GraphPad Prism 8. Data are expressed as the mean±SD, and two-tailed unpaired Student’s *t* tests were used to compare the differences between any two groups. One-way analysis of variance was used for comparisons among different groups. The survival curve was plotted using the Kaplan-Meier method and compared by the log-rank test. Differences were considered significant when *P*<0.05.

## Results

### Knockdown of *KLF5* enhances the cytotoxicity of olaparib

**Figure 1:**
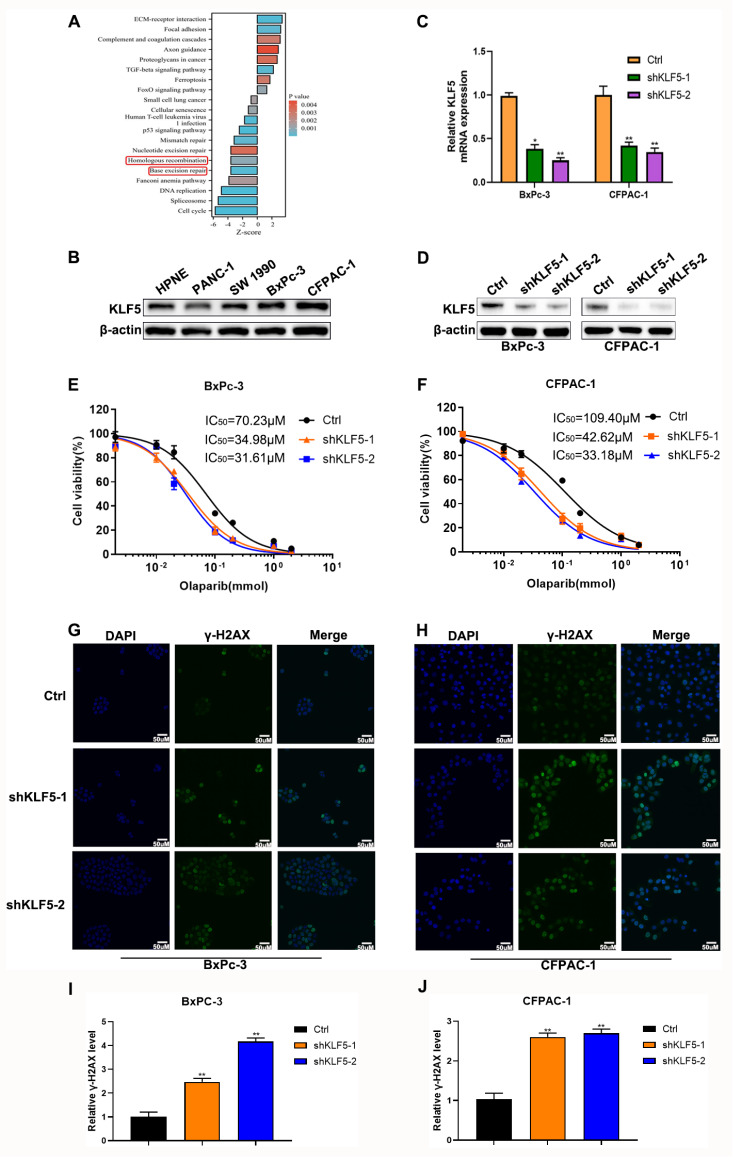
Figure 1 Knockdown of *KLF5* sensitives PDAC cells to olaparib (A) KEGG pathway enrichment. (B) Western blot analysis was used to investigate KLF5 expression in several cell lines. (C) qRT-PCR was used to explore the knockdown efficiency of shKLF5. (D) Western blot analysis was used to analyze the knockdown efficiency of shKLF5. (E,F) The curves depict the dose-dependent toxicity of olaparib in BxPC3 and CFPAC-1 cell lines transfected with shKLF5 or shRNA-NC. (G,H) Confocal microscopy suggested γ-H2AX (green) in KLF5-silenced PDAC cells that were pretreated with olaparib (100 μM) or not. (I,J) Relative γ-H2AX level in PDAC cells. *P<0.05, **P<0.01.

To further investigate the role of KLF5 in the sensitivity of olaparib in PDAC, we reanalyzed the previous transcriptome sequencing in which *KLF5* was knocked out in CFPAC-1 cells [34]. KEGG pathway analysis showed that inhibition of KLF5 significantly suppressed homologous recombination ( Figure 1A). We detected KLF5 protein levels in a human pancreatic duct epithelial cell line (hTERT-HPNE) and four pancreatic cancer cell lines to select suitable cell lines for further exploration. Our results indicated that KLF5 expression was higher in the BxPC-3 and CFPAC-1 cell lines ( Figure 1B). Thus, stable *KLF5*-silenced BxPC-3 and CFPAC-1 cells were established by infection with lentivirus and selected using puromycin. The knockdown efficiency was confirmed by qRT-PCR ( Figure 1C) and western blot analysis ( Figure 1D). To investigate whether KLF5 could decrease the IC _50_ of PARP inhibitors in pancreatic cancer cells, we examined the viability of cells cultured in olaparib at gradient concentrations. Abrogation of KLF5 could significantly sensitize BxPC-3 and CFPAC-1 cell lines to olaparib ( Figure 1E,F). Furthermore, we observed that suppression of KLF5 robustly increased the level of the DNA damage marker γ-H2AX induced by olaparib in BxPC-3 cells ( Figure 1G,I) and CFPAC-1 cells ( Figure 1H,J). These results demonstrated that KLF5 could regulate olaparib-induced cytotoxicity in pancreatic cancer cells.

### BRCA1 is positively correlated with KLF5 expression

**Figure 2:**
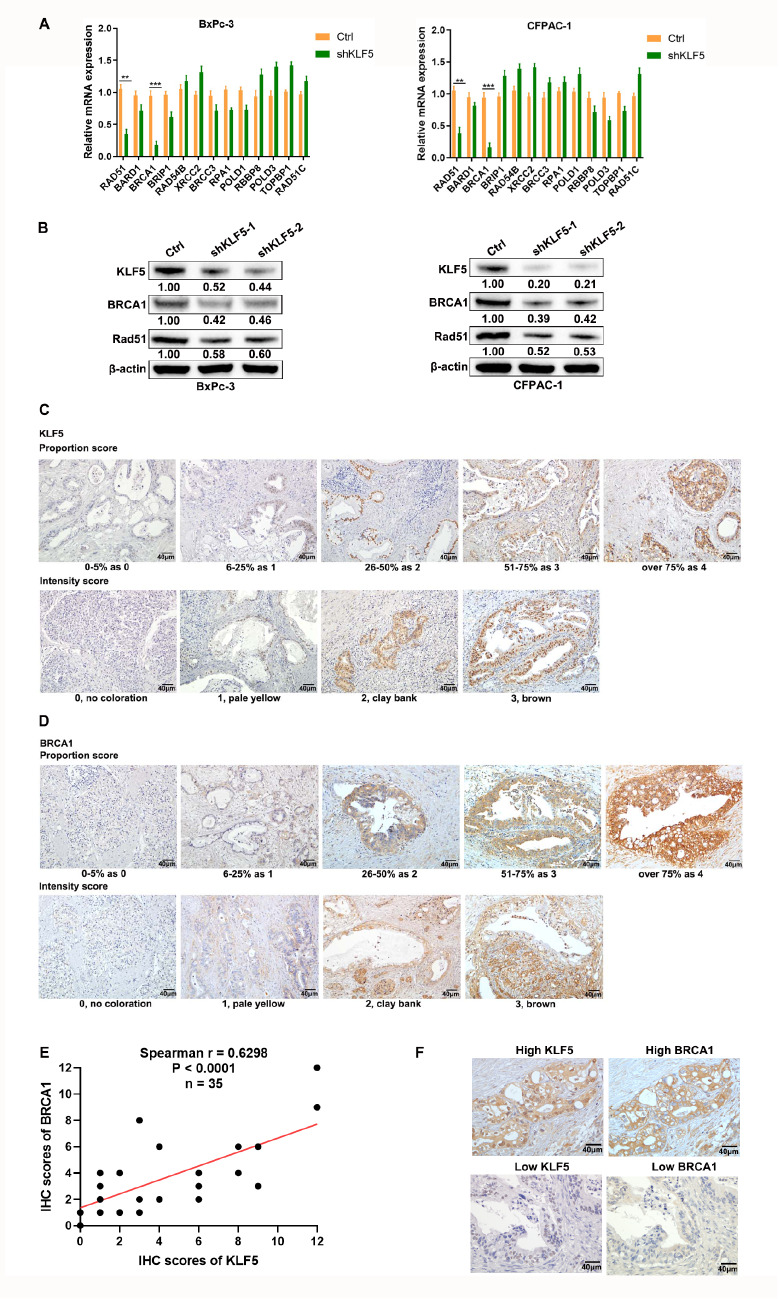
Figure 2 Correlation between the expression of KLF5 and homologous recombination-related genes (A) The mRNA levels of KLF5 and genes enriched in homologous recombination pathways in PDAC cells were measured by qPCR. (B) The protein levels of KLF5, BRCA1, and Rad51 were analyzed by western blot analysis. (C,D) Representative micrographs suggesting the intensity score and proportion score of KLF5 and BRCA1. (E) KLF5 was positively correlated with BRCA1 expression in pancreatic cancer patients, as indicated by IHC and scoring. (F) Patients with higher level of KLF5 showed higher BRCA1 expression. **P<0.01, ***P<0.001, ****P<0.0001.

There were 13 differentially expressed genes in the above homologous recombination pathway ( Figure 1A). qRT-PCR results suggested the most significant difference in BRCA1 expression, followed by Rad51 in BxPC-3 and CFPAC-1 cells ( Figure 2A), which was confirmed by western blot analysis ( Figure 2B). These results indicated that BRCA1 may be the key for KLF5 to regulate olaparib sensitivity. To further verify the results of the cell experiments, we investigated the correlation between BRCA1 and KLF5 in PDAC patients. The semiquantitative IHC scores of BRCA1 and KLF5 were obtained by multiplying the intensity scores and proportion scores. The representative expressions of BRCA1 and KLF5 are shown in Figure 2C,D. Then, we performed a statistical analysis of the relationship between BRCA1 and KLF5, and the results suggested that there was a robust positive correlation between BRCA1 and KLF5 in PDAC patients ( Figure 2E). Two typically positive examples of BRCA1 and KLF5 expression are shown in Figure 2F.

### *BRCA1* is a downstream target of KLF5

**Figure 3:**
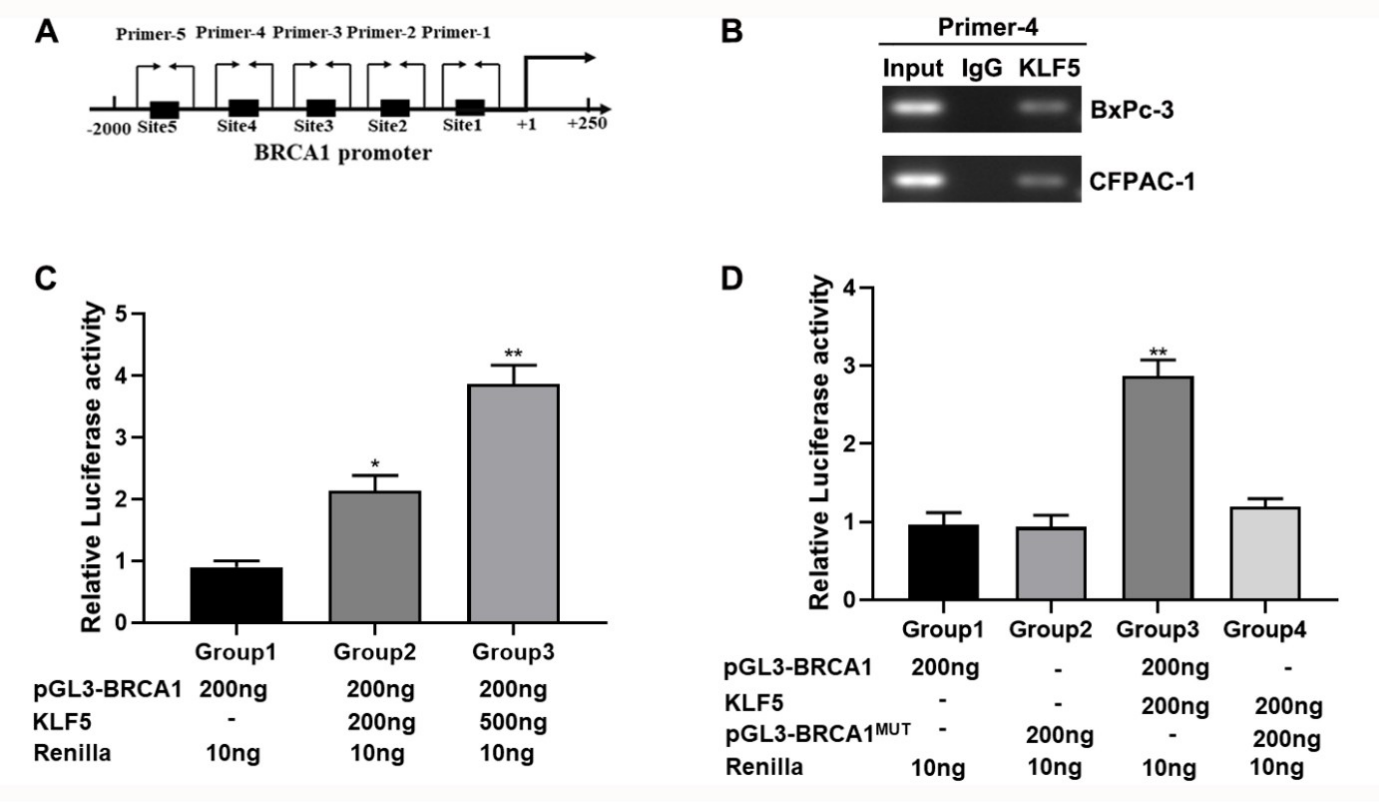
Figure 3 KLF5 is involved in BRCA1 transcription in PDAC (A) The position of the KLF5 binding sites in the BRAC1 promoter. (B) KLF5 binds to the sites of the BRAC1 promoter region in BxPC-3 and CFPAC-1 cells, as detected by ChIP assay. (C) KLF5 mediated BRAC1 promoter activity in HEK-293T cells. (D) KLF5 did not regulate mutated BRAC1 promoter activity in HEK-293T cells. *P<0.05, **P<0.01.

Based on the above results, we hypothesized that *BRCA1* may be a target of KLF5. We observed that KLF5 had several possible binding sites in the *BRCA1* promoter region ( Figure 3A). We then performed a ChIP assay with a KLF5 antibody to validate that KLF5 binds to the *BRCA1* promoter. The results suggested that KLF5 bound to the *BRCA1* promoter at the site of primer 4 ( Figure 3B) instead of the other four sites ( Supplementary Figure S1A). The subsequent luciferase assays indicated that regulation of KLF5 expression increased *BRCA1* promoter activity in a dose-dependent manner ( Figure 3C). These results were further invalidated by the mutation of BRCA1 binding sites in which the sequence was mutated from TCCCCTTCCC into GAAAAGGAAA ( Figure 3D).

### Overexpression of BRCA1 reverses enhanced cytotoxicity of olaparib induced by silencing of *KLF5*

**Figure 4:**
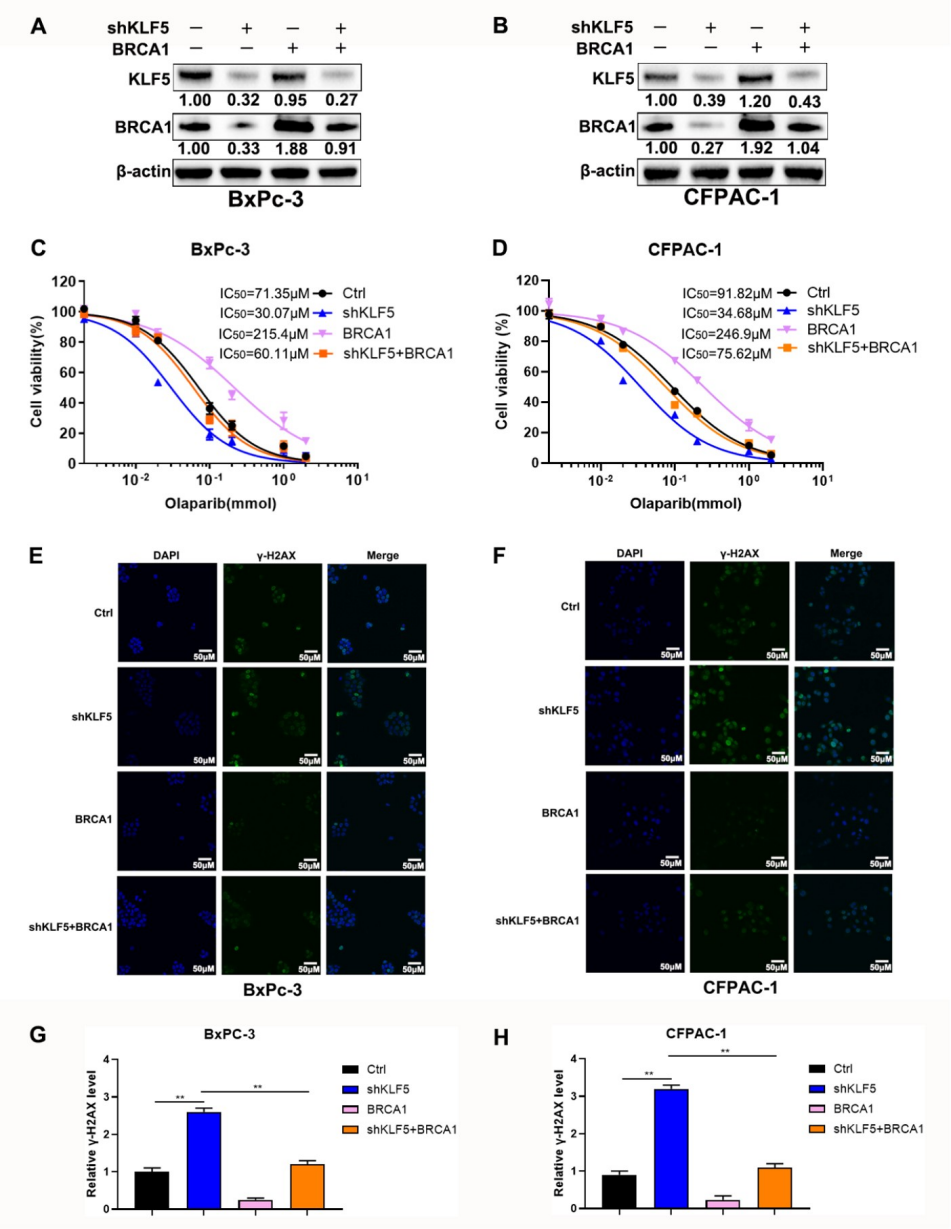
Figure 4 Overexpression of BRCA1 reverses increased sensitivity to olaparib induced by silenced KLF5 (A,B) Western blot analysis was utilized to measure the protein levels of KLF5 and BRCA1 in BxPC-3 and CFPAC-1 cells. (C,D) The CCK-8 assay depicted the dose-dependent toxicity of olaparib in BxPC3 and CFPAC-1 cell lines transfected with shKLF5 and BRCA1. (E,F) Confocal microscopy showed γ-H2AX (green) in BxPC-3 and CFPAC-1 cells that were pretreated with olaparib (100 μM) or not. (G,H) Relative γ-H2AX level in PDAC cells. **P<0.01

Our results suggested that KLF5 could mediate DNA damage and cytotoxicity induced by olaparib by regulating the expression of BRCA1. Subsequent western blot analysis suggested that overexpression of BRCA1 significantly reversed its decreased level caused by *KLF5* silencing in BxPC-3 and CFPAC-1 cells ( Figure 4A,B). Further results showed that increased BRCA1 significantly attenuated the enhanced cytotoxicity of olaparib induced by silencing of *KLF5* ( Figure 4C,D). However, overexpression of Rad51 could only slightly weaken the effect of inhibition of KLF5 ( Supplementary Figure S1B,C). The above results indicated that BRCA1 rather than Rad51 plays a critical role in KLF5-mediated sensitivity of pancreatic cancer cells to olaparib. Additionally, upregulation of BRCA1 impaired the increased DNA damage induced by the inhibition of KLF5 in BxPC-3 cells ( Figure 4E,G) and CFPAC-1 cells ( Figure 4F,H).

### KLF5 inhibition synergistically enhances PARP inhibitor activity

**Figure 5:**
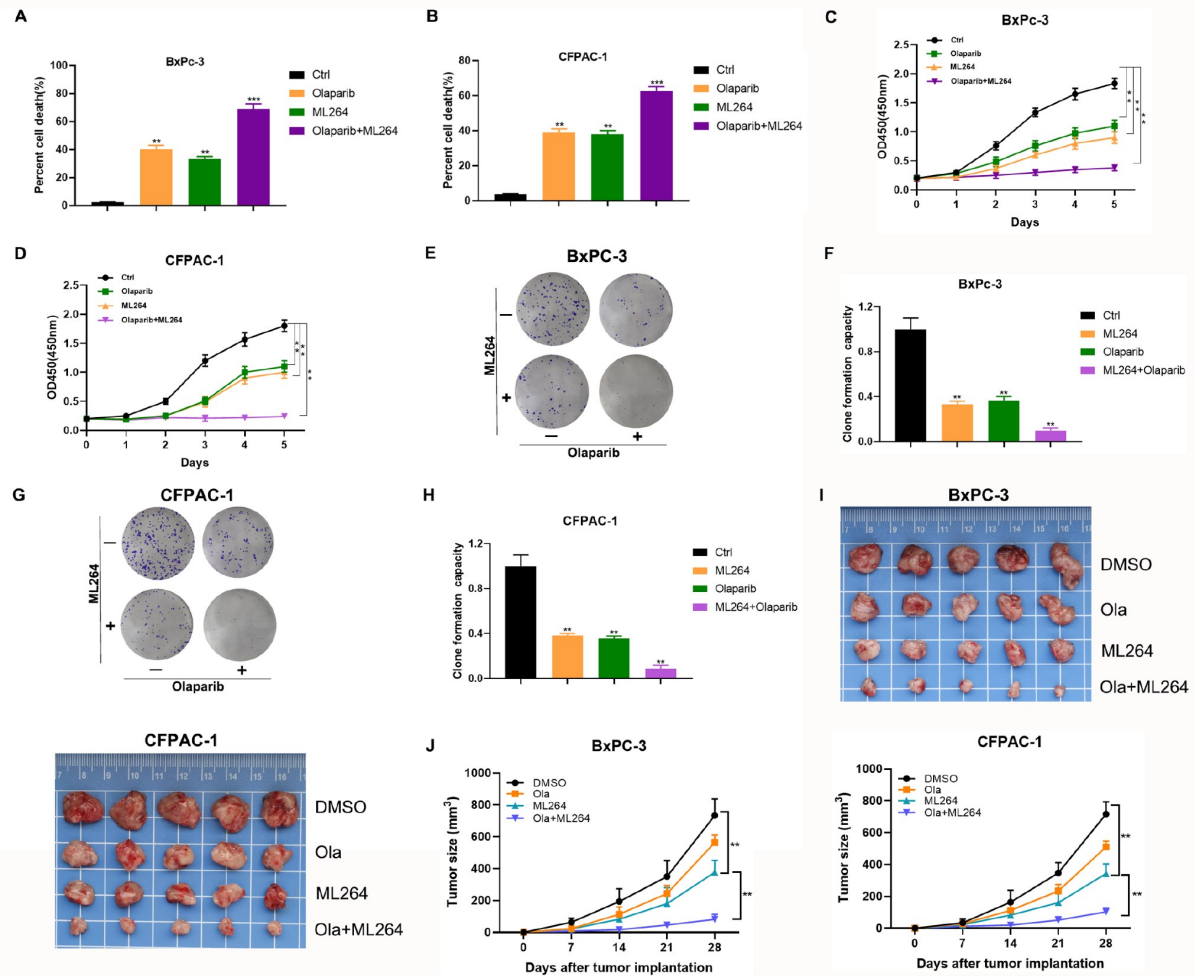
Figure 5 KLF5 inhibitors increase the sensitivity of PDAC cells to olaparib (A,B) Cell death was determined in BxPC-3 and CFPAC-1 cells treated with olaparib (100 μM) and ML264 (10 μM) using a CCK-8 assay. (C,D) The proliferation of BxPC-3 cells was evaluated in BxPC-3 and CFPAC-1 cells treated with olaparib and ML264 by CCK-8 assay. (E,F) The influence of olaparib and ML264 on BxPC-3 cells was determined by colony formation assay. (G,H) The influence of olaparib and ML264 on CFPAC-1 cells was measured by colony formation assay. (I) The mice were randomly divided into DMSO, olaparib, ML264, and olaparib+ML264 groups and treated as described in the Methods. (J) The tumor sizes were measured using Vernier calipers. Tumor growth curves were constructed based on the tumor volumes measured in the mice. **P<0.01, ***P<0.001.

The above results indicated that inhibition of KLF5 may sensitize PDACs to PARP inhibitors. Therefore, we sought to explore the pharmacological significance of inhibiting KLF5. As expected, olaparib or ML264 inhibited cell viability, and the lethal effect of olaparib combined with ML264 was much better than that of monotherapy ( Figure 5A,B). CCK-8 assay results indicated that olaparib or ML264 alone repressed cell growth, and the combination of the two inhibitors further decreased cell viability ( Figure 5C,D). Olaparib or ML264 alone suppressed colony formation in BxPC-3 and CFPAC-1 cells, and the addition of ML264 significantly enhanced the inhibitory effect of olaparib ( Figure 5E‒H).

We then established pancreatic xenograft tumor models to further determine whether inhibition of KLF5 sensitizes PDACs to PARP inhibitors *in vivo*. The combination of olaparib and ML264 significantly decreased the tumor growth rate and size when compared to monotherapy ( Figure 5I,J).

## Discussion

Considering the vital role of olaparib in maintenance treatment in patients with unresectable PDAC, developing a novel strategy inducing “BRCAness” could enable more patients to benefit clinically from PARP inhibitors. Our present results suggested that repression of KLF5 promoted olaparib-induced DNA damage in pancreatic cancer cells and increased olaparib sensitivity by suppressing the transcription of BRCA1, which could be reversed by upregulation of BRCA1. Furthermore, olaparib combined with an inhibitor of KLF5 showed stronger cytotoxicity to pancreatic cancer cells.

BRCAness refers to a phenotype of BRCA1/2 mutation. It depicts the state in which an HRR defect occurs in a tumor without a germline BRCA1/2 mutation [35]. The loss or mutation of genes such as *ATM*, *ATR*, *CDK12*, *CHEK2*, and *FANCA* involved in HR is also considered to induce BRCAness and may alter the therapeutic efficacy of PARP inhibitors and platinum [36]. Additionally, cancer cells inevitably develop resistance to PARP inhibitors. The most recognized explanation is the restoration of BRCA1/2 function by losing the frameshift caused by the original mutation and restoring the open reading frame [37]. Genetic reversal of genetic mutations could also result in the expression of full-length wild-type proteins [38].

Cancer cells can quickly obtain resistance to PARP inhibitors by losing hypermethylation of the *BRCA1* or *RAD51C* promoter and restoring mRNA and functional protein expression [ 39, 40]. Inhibition of BRCA1 may induce a BRCAness-like situation, which could significantly expand the clinical application and overcome potential resistance to PARP inhibitors.

Krüppel-like factors widely regulate cancer cell metastasis and proliferation, the tumor microenvironment, and cancer stem cells [41]. Based on the significant correlation between high expression of KLF5 and poor prognosis of pancreatic cancer [18], the vital role of KLF5 as a substrate of FBW7 was indicated by our previous study [16]. We explored whether KLF5 regulates the sensitivity to PARPis in pancreatic cancer. Our results indicated that abrogation of KLF5 facilitated DNA damage induced by olaparib and increased sensitivity to PARP inhibitors, which could be reversed by overexpression of BRCA1. Our study also revealed that the expression of Rad51 could be regulated modestly by KLF5, which is consistent with Sun’s research [18]. The above results suggested that inhibition of KLF5 may also induce BRCAness by downregulating Rad51.

Our investigation showed that BRCA1 could be regulated by KLF5 at the transcriptional level. As a key transcription factor, KLF5 mainly mediates the transcription of target genes by binding to specific regions of the promoter. *BRCA1* has multiple transcripts, and the most commonly used transcript sequence could contain over 5500 bp [42]. BRCA1/2 mutations include point mutations, small fragment insertions/deletions, and large fragment rearrangements [ 43, 44]. There are no mutation hotspots, and mutations could occur in the entire length of the *BRCA1*/ *2* gene compared with *EGFR*, *BRAF*, *Kra*s, etc. [ 43, 44]. Additionally, its expression level and protein activity are regulated by multiple mechanisms. Chen *et al* [45] discovered that miR-9 mediates the suppression of BRCA1 and hinders DNA damage repair in ovarian cancer cells. It has been reported that phosphorylated ETS transcription factors induced by the ERK pathway could repress the BRCA1 promoter [ 46, 47]. Moreover, phosphorylated ETS1 could downregulate the expression of BRCA1/2 [26]. It has been well established that the translated BRCA1 protein must be phosphorylated to achieve DNA damage repair function [ 48, 49]. BRCA1 can be phosphorylated by many kinases, such as CDK1, 2, casein kinase 2, and DNA damage-responsive kinases, including ATM, ATR, hCds1, and AKT [ 48‒ 53]. Johnson *et al*. [54] reported that suppression of CDK1 impairs the ability of cells to repair DNA. In addition, the prolyl isomerase Pin1 could maintain BRCA1 by preventing the ubiquitination of BRCA1 [55].

**Figure 6:**
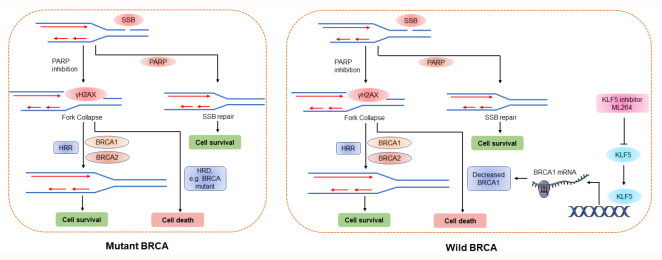
Figure 6 Schematic representation of the role of KLF5 in DNA damage repair and its underlying mechanism Downregulation of KLF5 significantly inhibits the expression of BRCA1 at the transcriptional level. Abrogation of KLF5 drives “BRCAness” and empowers PDAC cells with sensitivity to olaparib in BRCA1-proficient pancreatic cancer cells.

In conclusion, our study indicated that repression of KLF5 could render BRCA1-proficient pancreatic cancer cells BRCA1 deficiency and thus sensitized them to PARP inhibition. We also preliminarily investigated the underlying mechanism ( Figure 6).

## Supporting information

23385Supplementary_Table_S1

## Supplementary Data

Supplementary data is available at *Acta Biochimica et Biophysica Sinica* online.
