# Supplementary material for: Abrogation of KLF5 sensitizes BRCA1-proficient pancreatic cancer to PARP inhibition : KLF5 mediates sensitivity to PARP in BRCA1-proficient PDAC
Source: Acta Biochim Biophys Sin (Shanghai). 2024 Mar 4;56(4):576–85. doi: 10.3724/abbs.2023288 (PMC11090855; doi:10.3724/abbs.2023288)
Supplement: 23385Supplementary_Table_S1 [file 23385Supplementary_Table_S1.pdf]

Supplementary Table S1. Sequences of primers used in this study

Gene	Sequence (5′→3′)
RAD51 forward	CAACCCATTTCACGGTTAGAGC
RAD51 reverse	CAACCCATTTCACGGTTAGAGC
BARD1 forward	CTGCTCGCGTTGTACTAACAT
BARD1 reverse	TCCAATGCAGTCACTTACACAAT
BRCA1 forward	GAAACCGTGCCAAAAGACTTC
BRCA1 reverse	CCAAGGTTAGAGAGTTGGACAC
BRIP1 forward	GGAAACAGTCAAGAGTCATCGAA
BRIP1 reverse	TCTGAGCAATCTGCTTGTGTG
RAD54B forward	GCCAAACACTGATGATTTGTGG
RAD54B reverse	CCTGAGAAGAATGCGAGATAGC
XRCC2 forward	TGCTTTATCACCTAACAGCACG
XRCC2 reverse	TGCTTTATCACCTAACAGCACG
BRCC3 forward	GAGTCTGACGCTTTCCTCGTT
BRCC3 reverse	TGTATCATCGTTCAACTCCCCT
RPA1 forward	CGGGAATGGGTTCTACTGTTTC
RPA1 reverse	CGAGCACAAATGGTCCACTTG
POLD1 forward	CGGGGACGAGTTTGTGAAGA
POLD1 reverse	CGTATACGGAGTTGGCGCTC
RBBP8 forward	AGATCGGTTAAGAGCAGGCTT
RBBP8 reverse	TGCTGCCGGATATTTTCAAACT
POLD3 forward	ACCAACAAGGAAACGAAAACAGA
POLD3 reverse	GGTTCCGTGACAGACACTGTA
TOPBP1 forward	TTCAGCAACTCACAGTTAAGCA
TOPBP1 reverse	GGCACACTCATACTTCTGACC
RAD51C forward	TTTGGTGAGTTTCCCGCTGTC
RAD51C reverse	AACTTCTTTGCTAAGCTCGGAG
β-actin forward	GAGACCTTCAACACCCCAGC
β-actin reverse	GATAGCACAGCCTGGATAGCA
KLF5 forward	ACACCAGACCGCAGCTCCA
KLF5 reverse	TCCATTGCTGCTGTCTGATTTGTAG
ChIP forward 1	CAGAAGCAACCTGAACCCGA
ChIP reverse 1	CACGAGTCTCGGGCAAGTAG
ChIP forward 2	AAGCAACCTGAACCCGAACA
ChIP reverse 2	CGAGTCTCGGGCAAGTAGTC
ChIP forward 3	CCCAAACCCTCTTAGCGTGA
ChIP reverse 3	AACACTCGGAGAAACAGGGG
ChIP forward 4	AACCCTCTTAGCGTGACGTG
ChIP reverse 4	CACTCGGAGAAACAGGGGAC
ChIP forward 5	GCCCCTCTTCTTTGCAGGAT
ChIP reverse 5	TTGTTCGGGTTCAGGTTGC

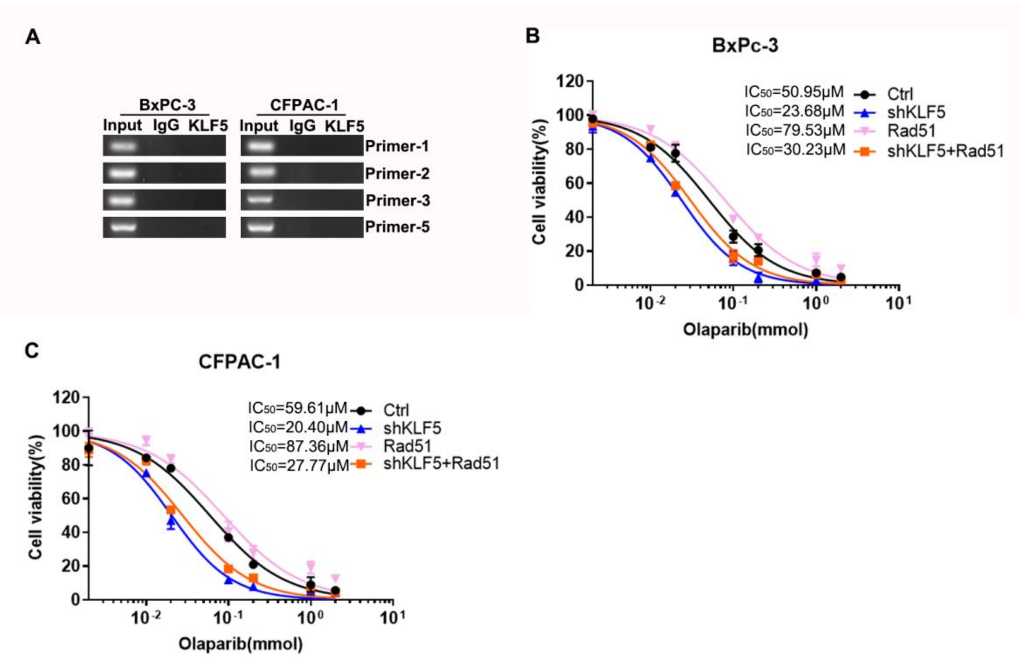

Supplementary Figure S1. The effects of KLF5 on BRCA1 and Rad51 (A) The binding of KLF5 to 4 predicted sites of the *BRAC1* promoter region in BxPC-3 and CFPAC-1 cells was detected by ChIP assay. (B,C) CCK-8 assay depicted the dose-dependent toxicity of olaparib in BxPC3 and CFPAC-1 cell lines transfected with shKLF5 and Rad51.
